# *In vitro* Synergy of Polyphenolic Extracts From Honey, Myrtle and Pomegranate Against Oral Pathogens, *S. mutans* and *R. dentocariosa*

**DOI:** 10.3389/fmicb.2020.01465

**Published:** 2020-07-24

**Authors:** Daniela Sateriale, Serena Facchiano, Roberta Colicchio, Chiara Pagliuca, Ettore Varricchio, Marina Paolucci, Maria Grazia Volpe, Paola Salvatore, Caterina Pagliarulo

**Affiliations:** ^1^Department of Science and Technology, University of Sannio, Benevento, Italy; ^2^Department of Molecular Medicine and Medical Biotechnology, University of Naples Federico II, Naples, Italy; ^3^Institute of Food Science – CNR, Avellino, Italy; ^4^CEINGE, Advanced Biotechnologies s.c.ar.l., Naples, Italy

**Keywords:** *in vitro* anticariogenic agents, polyphenolic extracts, oral pathogens, synergistic antimicrobial activity, *Streptococcus mutans*, *Rothia dentocariosa*

## Abstract

The increasing incidence rate of oral diseases, the wide spread of antimicrobial resistance, and the adverse effects of conventional antibiotics mean alternative prevention and treatment options are needed to counteract oral pathogens. In this regard, our study aims to evaluate the antibacterial activity of polyphenolic extracts prepared from acacia honey, myrtle leaves, and pomegranate peel against cariogenic bacteria, such as *Streptococcus mutans* and *Rothia dentocariosa*. The chemical-physical parameters of acacia honey and the RP-HPLC polyphenolic profile of pomegranate peel extract have been previously described in our studies, while the characterization of myrtle extract, performed by HPLC analysis, is reported here. All the extracts were used singly and in binary combinations to highlight any synergistic effects. Moreover, the extracts were tested in association with amoxicillin to evaluate their ability to reduce the effective dose of this drug *in vitro.* The values of minimal inhibitory concentrations and minimal bactericidal concentrations have been used to quantitatively measure the antibacterial activity of the single extracts, while the fractional inhibitory concentration index has been considered as predictor of *in vitro* anticariogenic synergistic effects. Finally, a time-kill curve method allowed for the evaluation of the bactericidal efficacy of the combined extracts. The microbiological tests suggest that acacia honey, myrtle, and pomegranate extracts are able to inhibit the cariogenic bacteria, also with synergistic effects. This study provides useful and encouraging results for the use of natural extract combinations alone or in association with antibiotics (adjuvant therapy) as a valid alternative for the prevention and treatment of oral infectious diseases.

## Introduction

Dysbiosis of oral microbiota, followed by the transition of several oral microorganisms from a commensal to pathogenic lifestyle, is a significant cause of dental caries (Nishikawara et al., 2007). Tooth decay is a biofilm-mediated oral disease that causes several disturbances in adults and children. If not treated in time, it can affect not only the mastication function, but also the individual’s speech, smile, and psychosocial environment, as well as their quality of life (Mathur and Dhillon, 2018). Even though its incidence rate has been reduced in recent years thanks to the use of preventive systems to maintain oral health, dental caries still remain one of the most prevalent chronic diseases, especially in the child population (Nishikawara et al., 2007). The etiology of dental caries is multifactorial; it includes the interplay between host factors, plaque bacteria, and diet. The current theory about the etiology of caries is based on the Ecological Plaque Hypothesis, according to which the plaque ecological balance is considered to be the key factor in determining an individual’s caries susceptibility (Marsh, 1991). The pivotal role of this theory is represented by dietary carbohydrates, which are metabolized by plaque bacteria to produce acid end-products, resulting in a drastic reduction of microenvironmental pH. This process, if prolonged, could determine an acidification of environments which would result in a drastic dissolution of minerals from the tooth structure. The relationship between plaque bacteria and tooth in the context of disease is highly complex and does not follow the classic exogenous infection model. Koch’s criteria, where an individual pathogen is implicated in a specific disease, are inapplicable to the polymicrobial biofilm-mediated caries disease (Caldwell et al., 1997; Sim et al., 2016). The bacteria associated with the caries disease have often been described as ‘opportunistic pathogens’; however, it has been suggested that since the bacteria implicated are resident bacteria, they should be described as pathobionts and not pathogens (Chow and Mazmanian, 2010; Ayres et al., 2012; Sim et al., 2016).

*Streptococcus mutans* is an acidogenic and aciduric bacterial species, considered as the primary pathogen in human dental caries (Van Houte, 1994; Tanzer et al., 2001). *S. mutans* is the dominant species in many, but not all, subjects with caries (Gross et al., 2012). Elevated levels of *S. salivarius*, *S. sobrinus*, and *S. parasanguinis* are also associated with caries, especially in subjects with no or low levels of *S. mutans* (Gross et al., 2012), suggesting that these species are alternative pathogens, and that multiple species may need to be targeted for anticariogenic interventions (Becker et al., 2002; Aas et al., 2008; Gross et al., 2010; Tanner et al., 2011). In other studies, when *S. mutans* was absent, additional species have been associated with caries disease, including *Lactobacillus* species, especially in the case of advanced caries (Aas et al., 2008; Gross et al., 2010).

Caries-related microorganisms also include *Propioni bacterium* (Aas et al., 2008) and *Rothia* spp. In particular, *R. dentocariosa* is involved in the cariogenic process, due to its ability to adhere to tooth surfaces, produce large amounts of acid, and contribute to biofilm formation (Nishikawara et al., 2007). *Rothia dentocariosa* is also associated with periodontal inflammatory disease (Kataoka et al., 2013) and it may turn up as the unexpected pathogen in serious human infections such as bacteremia, pneumonia, endocarditis, and peritonitis (Braden et al., 1999; Yang et al., 2009; Keng et al., 2012; Khan et al., 2014). Therefore, the pathobiology of dental caries is extremely complex and data from recent molecular microbiological studies have further defined the role of the oral microbiome in the etiology of dental caries. In addition to the contribution of classical cariogenic prokaryotes, such as *Streptococcus mutans*, in the etiology of caries, the hypothesis of the participation of the oral eukaryotic fungal pathogen *Candida* in the dental caries process is gaining consideration (Pereira et al., 2018).

Interactions occurring between oral microbiota, dental plaque, and the dental surface during dental caries represent scientific challenges. Likewise, the research for effective therapeutic and preventive tools represents a major challenge. The need for new anticariogenic agents has directed scientific interest toward searching for effective natural products against oral pathogens. Indeed, in the last few decades, there was a considerable increase in the use of natural sources for the development of preventive and therapeutic agents contributing to oral health care (Ferrazzano et al., 2013; Martínez et al., 2017). Herbal therapies and nutraceutical agents are used worldwide to treat altered health conditions. Since plants and other natural products, such as honey, are often the sources of novel drugs, the detailed scientific screening of their bioactive effects still remains a priority in the development of new antimicrobial agents (Lautié et al., 2008; Albaridi, 2019).

Honey has been used for several centuries in many countries as a treatment for several diseases, even before knowledge existed on the causes of infection (Albaridi, 2019). As a result, many studies have analyzed the composition of honey and have studied the physical and chemical properties that may give rise to its ability to counteract various microorganisms (Alvarez-Suarez et al., 2010; Albaridi, 2019). Recently, *in vitro* clinical trials have confirmed its broad antimicrobial spectrum, due to its acidity, the osmotic effect coming from the high concentration of sugars, and the presence of various factors, including hydrogen peroxide, lysozyme, flavonoids, and various peptides added by bees (Israili, 2014). Specifically, the most abundant polyphenols characterized in extracts obtained from honey, and other honeybee products, include quercetin, isorhamnetin, kampferol, caffeic acid, galangin, ferulic acid, and *p*-cumaric acid (Buratti et al., 2007).

Medicinal plants, such as *Myrtus communis* L., are a source of new compounds which can be used both in the food industry and for medical purposes, primarily as antimicrobial agents (Aleksic and Knezevic, 2014). Extracts obtained from several vegetal portions of *M. communis* L. are characterized by polyphenolic compounds, including phenolic acids, tannins, and flavonoids, which mediate antioxidant effects and have a remarkable activity against different harmful microorganisms (Rasooli et al., 2002; Akin et al., 2012). Some results have indicated that phenolic compounds significantly contribute to antibacterial activity (Shan et al., 2007; Aleksic and Knezevic, 2014).

In this frame, the aim of the present work has been to define the *in vitro* antibacterial profile of polyphenolic extracts of *Robinia pseudoacacia* honey and *Myrtus communis* leaves against two critical cariogenic bacteria, *Streprococcus mutans* and *Rothia dentocariosa*. The effects of binary combinations of acacia honey and myrtle leaves extract with each other and with pomegranate peel extract, whose *in vitro* anticariogenic efficacy has been previously characterized (Ferrazzano et al., 2017), and with amoxicillin, an antibiotic often used in the treatment of oral infections, are also investigated.

## Materials and Methods

### Preparation of Hydro-Alcoholic Polyphenolic Extracts From Honey, Myrtle and Pomegranate

The extracts for the microbiological assays were prepared from samples of fresh acacia honey (*Robinia pseudoacacia* L.), dry myrtle (*Myrtus communis* L) leaves, and dry pomegranate (*Punica granatum* L.) peel fruit, with solid-liquid solvent-extraction method. The dilution ratio was 1:10 (w/v). The procedure of the hydro-alcoholic polyphenolic extracts production of acacia honey was similar to that described by Sateriale et al. (2019), except for the extraction buffer, which was composed of ethanol and distilled water at 50% (v/v) instead of acidified water. The chemical-physical profile of *Robinia pseudoacacia* honey, including the concentration of polyphenols, flavonoids, and mineral elements, as well as the identification of major volatile molecules, was previously described in Colucci et al. (2016).

The fresh myrtle leaves used for the polyphenolic extraction were harvested from plants growing in the Cilento National Park and Vallo di Diano (Campania, Italy). After drying at 40°C in an incubator for 5 days, the leaves were fine-powdered using a mixing grinder. Leaf powder was mixed with a hydro-alcoholic buffer, composed of ethanol and distilled water at 50% (v/v), reaching a final concentration of 0.1 g/mL. The extraction method consisted of stirring the solid matrix into the selected solvent at room temperature for 30 min, using a rotary shaker. After extract centrifugation at 10000 rpm for 15 min at room temperature, the double supernatant filtration with Whatman N°1 filter paper and membrane filters Millex-GS (Merk-Millipore) with a porosity of 0.22 μm, was carried out.

The hydro-alcoholic polyphenolic extracts of pomegranate peels used in this study were prepared according to Pagliarulo et al. (2016). Briefly, the characterization of phenolic compounds was obtained by HPLC (High Performance Liquid Chromatography) and MALDI-TOF MS analysis.

### Determination of Polyphenols Content

The amount of total polyphenols in the hydro-alcoholic extracts from honey, myrtle, and pomegranate, was determined by a colorimetric assay of Folin-Ciocalteu, as reported in Picariello et al. (2016) and similarly to Sateriale et al. (2019). In brief, 0.5 mL of each extract and 2.5 mL of Folin reagent (0.2 mol L^–1^) were mixed. After storing each sample away from light for 5 min, 2 mL of 7.5% Na_2_CO_3_ were added to the mix. The incubation of samples for 2 h in the dark at room temperature was carried out and subsequently the absorbance was determined by spectrophotometry (UV/Vis-6715 Jenway Spectrophotometer) at a wavelength of 760 nm. The Gallic acid standard (Sigma-Aldrich Chemie, Steinheim, Germany) (0–200 mg L^–1^) was used to produce the calibration curve, in order to express the total phenolic content in mg of GAE for g of each sample. In particular, aliquots of three separately prepared extracts from each natural matrix were analyzed.

The identification and quantification of the main polyphenolic compounds in hydro-alcoholic extracts prepared from *Myrtus communis* L. leaves was performed using LC-4000 series integrated HPLC systems (JASCO, Tokyo, Japan). A C18 Luna column 5 μm particle size, 25 cm × 3.00 mm i.d. (Phenomenex, Torrance, CA, United States) was used. The mobile phase composed of water with 0.5% formic acid (v/v) (solvent A) and HPLC grade methanol (solvent B) was used and the linear gradient started with 5% B in A to reach 20% B in A at 5 min, 25% B in A at 45 min, 45% B in A at 47 min, 55% B in A at 57 min, and 80% B in A at 67 min, with some modifications to the Lorach method (Lorach et al., 2002). The flow rate of 0.8 mL min^–1^ was maintained, and the injection volume of the sample was 20 μL. The column temperature was 30°C. The composition was identified by comparing its retention time with standards (Gallic acid, Quercetin and Myricetin from Sigma) and literature data.

### Microorganisms and Microbial Growth

The *Streptococcus mutans* ATCC 25175 (LGC Standards, United Kingdom) strain, isolated from carious dentin, and the *Rothia dentocariosa* Rd1 clinical isolate from samples of dental plaque provided by the Pediatric Dentistry Department of “Federico II” University of Naples (Italy), where it was identified by MALDI-MS and BD Phoenix^TM^ systems as described in Ferrazzano et al. (2017), were used as test microorganisms in order to evaluate the *in vitro* anticariogenic activity of the hydro-alcoholic polyphenolic extracts. Bacteria were cultured in aerobic conditions at a temperature of 37°C in Brain Heart Infusion (BHI) agar/broth medium (Oxoid S.p.a., Milan, Italy) and on Columbia CNA agar medium (5% Sheep Blood) supplemented with Colistin and Nalidixic Acid (Oxoid S.p.a., Milan, Italy).

Bacterial strains were maintained at 4°C on agar media. The isolates were stored frozen at −80°C in BHI broth supplemented with 10% glycerol (v/v) (Carlo Erba Reagents, Milan, Italy) until use and the working cultures were activated in the respective broth at 37°C for 15–18 h.

### *In vitro* Anticariogenic Activity Assays of the Hydro-Alcoholic Polyphenolic Extracts

#### Agar Diffusion Method

To evaluate the *in vitro* anticariogenic effects of the hydro-alcoholic polyphenolic extracts from honey, myrtle, and pomegranate, individually and in binary combination, with or without amoxicillin, against cariogenic microorganisms, an *in vitro* antimicrobial activity assay was performed using the agar diffusion method, as reported in Bauer et al. (1966). Briefly, bacteria were grown in BHI broth until they reached an optical density (O.D.) of 0.5 at a wavelength of 600 nm. Then an aliquot of microbial suspension (200 μL) was spread on the agar media and paper disks (6 mm in diameter, Oxoid S.p.a., Milan, Italy) were impregnated with the extracts and placed on the media. Amoxicillin (AMX) (Aesculapius Farmaceutici Srl - Via Cefalonia 70, 25124, Brescia, Italy) was used as positive control, while the extraction buffer was used as a negative control. After plates’ incubation at 37°C for 24 h, the size of the observed inhibition zones was measured. The mean diameter of the inhibition zones (MDIZ) (expressed in mm) produced by the natural extracts, used alone and in combination, allowed for the expression of their *in vitro* anticariogenic activities against the tested microorganisms.

#### Tube Dilution Method

The susceptibility of cariogenic strains to different concentrations of single polyphenolic extracts and in binary combination, with or without AMX, was determined by the tube dilution method with standard inoculum 1^∗^10^5^ CFU/mL (Colonies Forming Units/mL), according to Clinical and Laboratory Standards Institute (CLSI) 2017 guidelines (Varaldo, 2002; CLSI, 2020). AMX was used as a positive control, while the hydro-alcoholic extraction buffer of extraction was used as a negative control. Minimum inhibitory concentration (MIC) was assigned to the lowest concentration of the single *in vitro* anticariogenic agent or binary combination of *in vitro* anticariogenic agents which prevents microbial growth. The minimal bactericidal concentration (MBC) was defined as the minimum concentration of agent or binary combination of *in vitro* anticariogenic agents that killed 99% of bacteria from the initial inoculum.

#### *In vitro* Synergistic/Antagonistic Activity of Acacia Honey, Myrtle Leaves and Pomegranate Peel Polyphenolic Extracts Binary Combinations and Amoxicillin

The effects of honey, myrtle, and pomegranate polyphenolic extracts, alone or in combination, with or without AMX, were deemed synergistic, indifferent, or antagonistic against the two oral pathogens, thanks to the measuring of the fractional inhibitory concentration index (FICI) of binary combinations. In particular, the following formulae, in accordance with the Odds’ interpretation (Odds, 2003), were used. In brief: Fractional Inhibitory Concentration (FIC) = MIC of antimicrobial agent in the binary combination/MIC of single antimicrobial agent; FICI = FIC of antimicrobial agent 1 + FIC of antimicrobial agent 2. In our study, the FIC was defined as the minimum inhibitory concentration (MIC) of the polyphenolic extract used in combination, divided by the MIC of the same extract used alone. The FICI was defined as the sum of the FICs for each binary combination and expresses the type of interaction of the different agents used as anticariogenic *in vitro* (particularly, FICI ≤ 1, synergy; FICI > 1 or ≤ 4, indifference; FICI > 4, antagonism).

#### Time-Killing Assay With Binary Combination of Myrtle Leaves and Pomegranate Peel Polyphenolic Extracts

To verify the inhibitory synergistic effect of the binary combination of pomegranate and myrtle polyphenolic extracts on the survival of *S. mutans* and *R. dentocariosa*, a time-killing assay was performed, which allowed for the investigation of the bactericidal efficacy of this antimicrobial combination over time. In particular, an assay of bacterial survival, with single and mixed cultures of two microorganisms, was carried out, with increasing concentrations of the binary combination of the two extracts in a 1:1 ratio (0, 5, 10, 20, 30 μg μL^–1^). To evaluate the survival of each strain, during the observation period of 168 h, aliquots of serial dilutions of the bacterial suspensions were spread on BHI agar, and the plates were incubated at 37°C for 48 h to evaluate the viable bacterial colony counts.

### Statistics

Three replicates were carried out for all analyses and the antimicrobials tests were performed with independent microbial cultures. Results were graphically reported by “GraphPad Prism 6” software and statistical significance was validated by parametric and non-parametric tests. In particular, the TPC (Total Polyphenol Content) data were analyzed using a Mann–Whitney test comparing two groups at a time. The two-way ANOVA test was used to perform significance analysis of Log CFU (Colony Forming Unit) data, with Bonferroni *post hoc* correction for multiple comparisons against a control, indicating the statistical significance of treated bacterial cultures with extracts against monitored bacterial cultures in absence of the extracts. Statistical significance was assigned to all *P*-values < 0.05.

## Results

### Characterization of the Extracts

**FIGURE 1:**
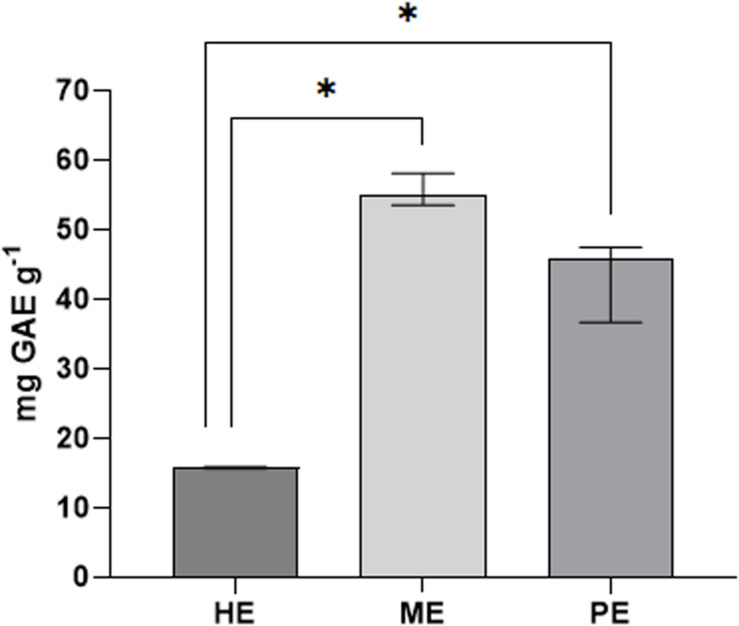
Total polyphenols of honey, myrtle, and pomegranate hydro-alcoholic extracts. The amount of total phenols in the extracts was estimated by Folin-Ciocalteu assay, expressed in mg of gallic acid equivalents (GAE) per g of originary solid matrix. PE, pomegranate extracts; ME, myrtle extracts; HE, honey extracts. Results are indicated as median with error bars of data obtained by triplicate experiments. Asterisks indicate statistical significance examined by Mann–Whitney test for multiple reciprocal comparison among the extracts (^∗^*P* < 0.1).

The total polyphenol content (TPC) in honey, myrtle, and pomegranate extracts is reported in Figure 1, indicated as mg of gallic acid equivalents (GAE) for g of sample. The polyphenol amount was found to be 15.75 mg GAE g^–1^ for acacia honey extracts, 54.86 mg GAE g^–1^ for myrtle leaves extracts, and 45.9 mg GAE g^–1^ for pomegranate peel extracts.

The HPLC profile of myrtle leaves extract is reported in Supplementary Figure S1, at wavelengths of 280 nm. The different absorption characteristics have been exploited to distinguish total phenols at 280 nm. Peaks comparison was performed on the basis of standard analysis and literature data (Romani et al., 1999, 2004; Yoshimura et al., 2008). The most abundant components were Galloyl derivatives and tannins as phenolic acids, and Myricetin and Quercetin derivatives as flavonols as reported by Romani et al. (2004).

### *In vitro* Anticariogenic Activity of Acacia Honey, Myrtle Leaves, and Pomegranate Peel Polyphenolic Extracts Against *S. mutans* ATCC 25175 and *R. dentocariosa* Rd1 Strains

Hydro-alcoholic polyphenolic extracts from honey, myrtle, and pomegranate exhibited an appreciable inhibitory activity against both *S. mutans* ATCC 25175 and *R. dentocariosa* Rd1 strains, as demonstrated by the evident inhibition zone of bacterial growth estimated through the agar diffusion method. The mean diameters of the inhibition zones (MDIZ) of bacterial growth exerted by the extracts, used individually and in binary combination, with or without amoxicillin (AMX), against the two cariogenic bacteria was reported in Table 1. The MDIZ ranged between 7.5 ± 0.5 mm (honey extracts, 1 mg/disk, vs. *S. mutans*) and 29.0 ± 1.9 mm (AMX, 50 μg/disk, in binary combination with myrtle extracts, 1 mg/disk, vs. *R. dentocariosa*). AMX, tested as a positive control, showed antibacterial efficacy against both cariogenic isolates, while no effects were observed for the negative control, i.e., the hydro-alcoholic buffer used for the extraction.

**TABLE 1:** *In vitro* anticariogenic activity of honey, myrtle, and pomegranate polyphenolic extracts, and amoxicillin, used individually and in binary combinations, determined with the agar diffusion method.

Antibacterial agents	MDIZ (mm)	
	
	*S. mutans* ATCC25175	*R. dentocariosa* Rd1
PE (2 mg/disk)	16.2 ± 1.0	15.7 ± 0.6
ME (2 mg/disk)	19.8 ± 2.2	17.2 ± 0.6
HE (2 mg/disk)	10.0 ± 0.8	12.0 ± 0.8
PE (1 mg/disk)	11.2 ± 0.2	12.8 ± 0.2
ME (1 mg/disk)	10.5 ± 0.4	13.5 ± 0.4
HE (1 mg/disk)	7.5 ± 0.5	08.2 ± 0.6
PE (1 mg/disk) + ME (1 mg/disk)	20.7 ± 2.0	19.2 ± 1.3
ME (1 mg/disk) + HE (1 mg/disk)	19.5 ± 1.8	17.3 ± 0.6
HE (1 mg/disk) + PE (1 mg/disk)	16.3 ± 0.8	16.1 ± 0.8
AMX (100 μg/disk)	26.2 ± 1.4	28.5 ± 2.0
AMX (50 μg/disk)	23.7 ± 2.6	25.8 ± 1.0
AMX (50 μg/disk) + PE (1 mg/disk)	26.5 ± 1.6	28.7 ± 2.2
AMX (50 μg/disk) + ME (1 mg/disk)	26.8 ± 1.5	29.0 ± 1.9
AMX (50 μg/disk) + HE (1 mg/disk)	24.0 ± 0.8	23.8 ± 1.6

The inhibition zone (mm) is reported as mean of triplicate assay ± standard deviation. MDIZ, mean diameter inhibition zone; PE, pomegranate extracts; ME, myrtle extracts; HE, honey extracts; AMX, amoxicillin.

The *in vitro* anticariogenic activity of the extracts was also confirmed by quantitative assays. The minimum inhibitory concentration (MIC) and minimum bactericidal concentration (MBC) values are reported in Table 2. Hydro-alcoholic polyphenolic extracts from pomegranate, myrtle, and honey showed bacteriostatic and bactericidal effects against both cariogenic strains. The inhibitory effect of extracts on cariogenic bacteria is evident both when used individually and in binary combination.

**TABLE 2:** Quantitative evaluation of *in vitro* anticariogenic activity of acacia honey extract, myrtle leaves extract, and pomegranate peel extract, and amoxicillin, used individually and in binary combinations.

Antibacterial agents	*S. mutans* ATCC25175		*R. dentocariosa* Rd1	
		
	MIC (μ g/μ L)	MBC (μ g/μ L)	MIC (μ g/μ L)	MBC (μ g/μ L)
PE	10.00	25.00	10.00	15.00
ME	10.00	20.00	10.00	30.00
HE	15.00	35.00	15.00	30.00
PE + ME	5.00	20.00	5.00	25.00
ME + HE	10.00	20.00	10.00	15.00
HE + PE	10.00	25.00	10.00	30.00
AMX	0.05	1.00	0.03	0.60
AMX + PE	0.03	1.00	0.02	0.80
AMX + ME	0.03	1.20	0.02	0.80
AMX + HE	0.05	1.00	0.03	0.60

*PE, pomegranate extracts; ME, myrtle extracts; HE, honey extracts; AMX, amoxicillin. MIC, minimum inhibitory concentration; MBC, minimum bactericidal concentration.*

### *In vitro* Anticariogenic Effects of Binary Combinations of Acacia Honey, Myrtle Leaves, and Pomegranate Peel Polyphenolic Extracts, and Amoxicillin, on *S. mutans* ATCC 25175 and *R. dentocariosa* Rd1 Strains

The *in vitro* anticariogenic activity of the binary combination of acacia honey, myrtle leaves, and pomegranate peel extracts, with or without AMX, was measured by the determination of fractional inhibitory concentration (FIC) value, for each extract and for the AMX, and of the FIC index (FICI) for each binary combination. FIC and FICI values are reported in Table 3. The binary combinations of pomegranate/myrtle extracts, AMX/pomegranate extracts, and AMX/myrtle extracts showed synergistic *in vitro* anticariogenic effects against both *S. mutans* and *R. dentocariosa* (Table 3).

**TABLE 3:** Antibacterial effects of binary combinations of pomegranate, myrtle, honey extracts, and amoxicillin on *S. mutans* and *R. dentocariosa.*

*Bacterial isolates*	*Binary combinations*	*Individual FIC*	*FIC index (FICI)*	*Interaction interpretation*
*S. mutans* ATCC25175	PE + ME	0.500/0.500	=1	*Synergy*
	ME + HE	1.000/0.667	>1	*Indifference*
	HE + PE	0.667/1.000	>1	*Indifference*
	AMX + PE	0.600/0.003	<1	*Synergy*
	AMX + ME	0.600/0.003	<1	*Synergy*
	AMX + HE	1.000/0.333	>1	*Indifference*
*R. dentocariosa* Rd1	PE + ME	0.500/0.500	=1	*Synergy*
	ME + HE	1.000/0.667	>1	*Indifference*
	HE + PE	0.667/1.000	>1	*Indifference*
	AMX + PE	0.667/0.002	<1	*Synergy*
	AMX + ME	0.667/0.002	<1	*Synergy*
	AMX + HE	1.000/0.002	>1	*Indifference*

*PE, pomegranate extracts; ME, myrtle extracts; HE, honey extracts; AMX, amoxicillin; FIC, fractional inhibitory concentration; FICI, fractional inhibitory concentration index. FICI ≤ 1, synergy; FICI > 1 or ≤4, indifference; FICI > 4, antagonism.*

The interaction between the other binary combinations was indifferent, that is they were neither synergistic nor antagonistic, as shown by the results from the values of FIC index reported in Table 3.

### Synergistic Inhibitory Effect of Binary Combination of Pomegranate Peel and Myrtle Leaves Polyphenolic Extracts on the Survival of *S. mutans* ATCC 25175 and *R. dentocariosa* Rd1

**FIGURE 2:**
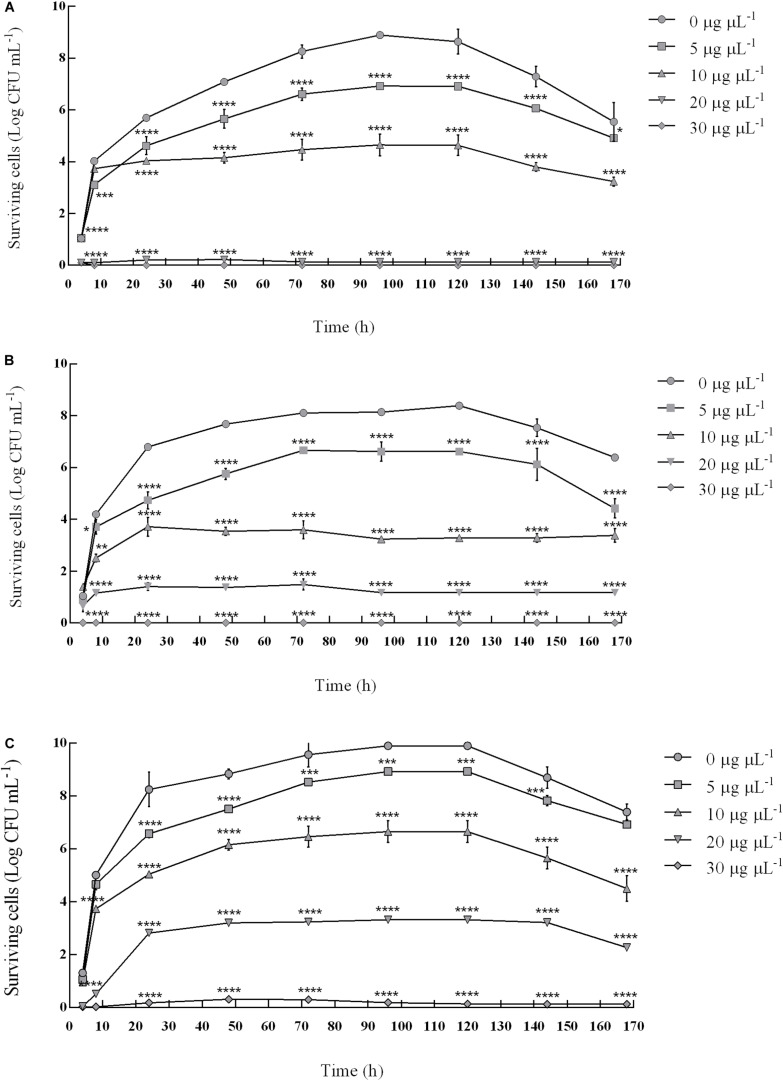
Synergistic inhibitory effect of binary combinations of pomegranate and myrtle extracts on the fitness of *S. mutans* ATCC 25175 and *R. dentocariosa* Rd1. Figure shows the survival of *S. mutans* ATCC 25175 in pure culture **(A)**, *R. dentocariosa* Rd1 in pure culture **(B)** and in mixed culture (*S. mutans* + *R. dentocariosa*) **(C)**, in absence and in the presence of 5 μg μL^–1^, 10 μg μL^–1^, 20 μg μL^–1^, and 30 μg μL^–1^ of pomegranate peel polyphenolic extracts in combination with myrtle leaves polyphenolic extracts (PE + ME), added in a 1:1 ratio. The experiments were performed in triplicate and statistical significance was examined by the Two-way ANOVA test with a Bonferroni correction for multiple comparisons against the control. Results are indicated as means ± SDs. Asterisks (*P < 0.05; **P < 0.01; ***P < 0.001; ****P < 0.0001) indicate statistical significance of treated cultures against control ones, i.e., monitored bacterial cultures in absence of extracts.

Since the combination of pomegranate peel and myrtle leaves polyphenolic extracts showed a synergistic inhibitory activity against *S. mutans* ATCC 25175 and *R. dentocariosa* Rd1 clinical isolates (Table 3), the effects of this binary combination on their fitness were evaluated (Figure 2).

The assay highlighted that the survival of both bacterial isolates was compromised in a dose-correlated way by the binary combination of pomegranate and myrtle extracts. This combination showed bacteriostatic and bactericidal effects during the 168 h-observation time against the pure bacterial cultures of *S. mutans* and *R. dentocariosa*, and also against the mixed bacterial cultures of *S. mutans* and *R. dentocariosa*.

## Discussion

In recent years, dietary polyphenols have been successfully evaluated as preventive chemo-preventive and therapeutic agents (Barbieri et al., 2017) thanks to their direct antimicrobial action and antibiotic modulation activity (Stermitz et al., 2000; Gibbons, 2005).

Our *in vitro* microbiological assays demonstrated that hydro-alcoholic polyphenolic extracts of *Robinia pseudoacacia* (acacia) honey, *Myrtus communis* leaves, and *Punica granatum* L. fruit peel are able to counteract cariogenic bacteria of dental plaque. In particular, the extracts showed inhibitory activity against *S. mutans* ATCC 25175 and *R. dentocariosa* Rd1 clinical isolates, which are considered main etiological agents of dental caries. Our results are in agreement with several studies that demonstrated how polyphenols can slow down the growth of pathogenic microorganisms, affect the external morphology and membrane integrity, or inhibit some cellular processes that are involved in microbial growth (Daglia, 2012; Canonico et al., 2014).

In this study, the hydro-alcoholic extracts of myrtle leaves showed the highest content of total polyphenols, compared to pomegranate and honey extracts. Alcoholic and aqueous extracts prepared from myrtle leaves have been defined as rich sources of polyphenols (Amensour et al., 2009) and a content of total polyphenols higher than 20 mg GAE for g of dry sample could be considered as highly significant (Tawaha et al., 2007). Gallic acid derivatives, tannins, Myricetin, and Quercetin derivatives resulted in the most abundant phenolic compounds in hydro-alcoholic extracts prepared from *Myrtus communis* leaves, as confirmed by other literature data (Romani et al., 1999, 2004; Yoshimura et al., 2008). In particular, tannins are traditionally associated with high antibacterial activity against pathogens bacteria (Ferreira et al., 2012; Widsten et al., 2014). Hydro-alcoholic, methanolic, and ethanolic extracts from myrtle leaves and berries demonstrated high antibacterial activity, especially when they were tested against foodborne pathogens (Amensour et al., 2010). The quantitative evaluation of the antibacterial effect of the myrtle leaves extract tested in this study showed results (MBC was 20.00 μg mL^–1^ vs. *S. mutans* and 30.00 μg mL^–1^ vs. *R. dentocariosa*) in line with literature data. An experimental study investigated the antibacterial effects of myrtle extracts against several human pathogens by dilution methods. Particularly, the myrtle MBC was 0.5 mg mL^–1^ for *S. aureus*, 2.5 mg mL^–1^ for *Proteus mirabilis* and *Proteus vulgaris*, 15 mg mL^–1^ for *Klebsiella* spp. and *Salmonella typhi*, and 20 mg mL^–1^ for *Pseudomonas aeruginosa*. The MBC of myrtle for the two relatively least sensitive species, *Shigella* sp. and *E. coli*, was 40 mg mL^–1^ and 45 mg mL^–1^ (Alipour et al., 2014). These data reflected the strong antibacterial activity of this herb (Alem et al., 2008). Moreover, in a recent paper, the antimicrobial activity of *Myrtus communis* oil (MCO) on some oral pathogens, including *S. mutans*, has been determined. The MICs of MCO for *S. pyogenes*, *S. mutans*, *C. albicans*, and *P. gingivalis* were 29.68 ± 4.8, 31.25 ± 0, 46.9 ± 16, and 62.5 ± 0 μg mL^–1^, respectively (Fani et al., 2014).

Also, the hydro-alcoholic extracts from pomegranate peel were rich in total polyphenols. In particular, according to our previous results, pedunculagin 1, punicalin, ellagic acid hexose, ellagic acid pentose, and ellagic acid deoxyhexose are the most abundant phenolic compounds in hydro-alcoholic pomegranate peel extracts (Pagliarulo et al., 2016). The same extracts had already been tested against *Staphylococcus aureus* and *Escherichia coli* clinical isolates, showing high inhibitory effects on their growth and survival (Pagliarulo et al., 2016), and also demonstrated *in vitro* antibacterial activity against cariogenic bacteria (Ferrazzano et al., 2017).

Finally, hydro-alcoholic extracts prepared from acacia honey showed the lowest content of total polyphenols compared to myrtle and pomegranate. However, these results are in agreement with those reported for aqueous extracts prepared from *Robinia pseudoacacia* honey of the same botanical origin (Colucci et al., 2016). In particular, the high concentration of flavonoids (2.41 ± 0.10 mg QE 100 g^–1^), as well as the low pH (3.5) of acacia honey, contribute to its antimicrobial activity (Colucci et al., 2016), but with weaker antibacterial effects against oral pathogenic species, like *S. mutans* and *R. dentocariosa*, with respect to myrtle and pomegranate hydro-alcoholic extracts.

From our results, the polyphenolic content of extracts seemed to be related to their antimicrobial activity. From a preliminary screening, obtained by agar diffusion method, it was shown that the growth of *S. mutans* and *R. dentocariosa* was strongly inhibited by pomegranate and myrtle polyphenolic extracts compared to the acacia honey polyphenolic extract. However, the *in vitro* anticariogenic effect of honey, myrtle, and pomegranate polyphenolic extracts showed MIC values similar to those obtained with polyphenols from different sources (*Prunus mume*, *Psidium guajava*, and *Polygonum cuspidatum*) against oral microorganisms (Prabu et al., 2006; Song et al., 2006). Notably, the binary combinations of some extracts seemed to have greater *in vitro* anticariogenic efficacy compared to that obtained with the single extract. Particularly interesting was the effect exerted by myrtle extracts in combination with pomegranate extracts that synergistically interfered with the bacterial growth, survival, and fitness of both *S. mutans* and *R. dentocariosa* strains, in pure and mixed bacterial cultures with time-lasting effects. In the literature, there are many studies regarding the characterization of the polyphenolic profiles of many natural extracts and on their antimicrobial properties, but, to our knowledge, no relevant study highlights the positive or negative effects deriving from possible combinations. The hypothesis that the synergistic antibacterial effects of extracts depend on their different polyphenolic content and relative mechanisms of action is among the most probable. In particular, the antibacterial effects of tannins is linked to their ability to modulate microbial metabolic pathways, to inhibit specific enzymes and membrane structural proteins, and to interfere with bacterial proliferation process (Scalbert, 1991; Omojate et al., 2014). Gallic acid is able to cause different changes in bacterial membrane properties, including hydrophobicity, charge, intra and extracellular permeability, and physicochemical properties, with consequent leakage of essential intracellular constituents (Borges et al., 2013). Moreover, inhibiting protein synthesis seems to be among the mechanisms of antibacterial activity of some myrtle polyphenols, such as myricetin, against gram-positive bacteria, such as *S. aureus*, and gram-negative bacteria, such as *B. cepacia* and *K. pneumoniae* (Xu and Lee, 2001). Regarding the mechanism of action linked to antimicrobial activity of polyphenolic extracts of pomegranate peel, it implicates the precipitation of membrane proteins, causing microbial cell lysis (Ismail et al., 2012).

In the last three decades, hundreds of research articles have been published on the antibacterial activity of phytochemicals and on their probable mechanisms of action (Hannah et al., 1989; Izzo et al., 1995; Pawar and Pal, 2002; Daglia, 2012; Erdem et al., 2015; Mahmoud et al., 2016; Nabavi et al., 2015a, b). Phytocompounds from different natural sources have shown a wide spectrum of action against gram-positive human pathogenic species, like *Staphylococcus aureus* and *Listeria monocytogenes* (Mansouri et al., 2001), which are among the most important etiological agents of foodborne diseases, and against Gram-negative bacterial species, such as *Escherichia coli* (Taheri et al., 2013) and *Bartonella henselae* (Ma et al., 2019), a facultative intracellular bacteria related to a higher resistance to the infection of other common pathogens in humans (Sommese et al., 2012). In addition, since phytochemicals cannot be used in monotherapy due to their higher MIC (100–5000 g mL^–1^) when compared with antibiotics (0.031–512 g mL^–1^), in the past few years the effects of combinations between antibiotics and phytochemicals have been studied. Several scientific evidences showed that phytochemicals can modulate bacterial mechanisms of antimicrobial resistance (Barbieri et al., 2017), suggesting their potential use in combination with antibiotics to decrease effective doses and increase antimicrobial activity (Touani et al., 2014; Santiago et al., 2015).

In our study, results obtained from the use of hydro-alcoholic polyphenolic extracts in combination with amoxicillin (AMX) were particularly remarkable. Amoxicillin is not only heavily used in medicine but is also frequently prescribed in dentistry (Tallo, 2016), together with other commonly used antibiotics against oral infections, such as tetracycline, minocycline, doxycycline, erythromycin, clindamycin, ampicillin, amoxicillin, and metronidazole, that have been extensively evaluated for treatment of periodontal diseases (Kapoor et al., 2012). In addition, oral streptococci showed a high susceptibility to amoxicillin, corresponding to 90% for *S. mutans* (Kariminik and Motaghi, 2015). Both pomegranate and myrtle extracts were shown to modulate the activity of AMX. The concentration of the antibiotic able to inhibit the *in vitro* growth of the two cariogenic bacteria were shown to be reduced. Numerous studies have reported that the use of combined therapies of botanical extracts and antibiotics are able to determine a significant reduction of the MICs of antibiotics against several pathogens (Betoni et al., 2006; Esimone et al., 2006; Sibanda et al., 2010).

The demonstrated ability of plant extracts to act in synergy with antibiotics could represent an effective approach in solving the problem of antimicrobial resistance (Nagoshi et al., 2006).

Results of our *in vitro* microbiological tests showed a strong anticariogenic effect of the hydro-alcoholic polyphenolic extracts from acacia honey, myrtle leaves, and pomegranate peel, alone and in synergy, against *S. mutans* and *R. dentocariosa* strains.

Although this study is limited to the analysis of these effects against only two of the main bacterial species that compose the complex oral plaque community, these data encourage the promotion of *in vitro* studies on biofilm models for the possible development of natural medical tools in the treatment and prophylaxis of dental caries. Furthermore, the synergistic activity of myrtle and pomegranate extracts in combination with AMX suggests that the natural extracts can be used as adjuvants of conventional antibiotic therapy and as an alternative therapeutic strategy against oral pathogens. Therefore, the polyphenols from honey, myrtle, and pomegranate represent nutraceutical agents to recommend in the prevention and treatment of oral diseases as a valid alternative to synthetic drugs, especially if they are detrimental and ineffective. This knowledge opens up a wide range of possibilities for the combined antimicrobial therapy against cariogenic bacteria and other oral pathogens (Zacchino et al., 2017). More research is needed to define in detail the antimicrobial activity played by these natural extracts. Future studies, such as *in vivo* testing and clinical trials, could allow the development of new antimicrobials as alternative agents in combination with existing antibiotics in clinical use, taking another step forward to overcome the problem of the antimicrobial resistance phenomenon.

## Data Availability Statement

The datasets generated for this study are available on request to the corresponding author.

## Author Contributions

DS and CaP conceived and planned the work. SF played part to experimental carrying out. RC, ChP, EV, MP, MV, and PS contributed to the design and implementation of the research, to the analysis of the results, and to the writing of the manuscript. All the authors agree to be accountable for the content of the manuscript.

## Conflict of Interest

The authors declare that the research was conducted in the absence of any commercial or financial relationships that could be construed as a potential conflict of interest.

## Supplementary Material

FIGURE S1 Chromatographic profile, acquired at 280 nm, of *Myrtus communis* L. leaves hydro-alcoholic extract.
