# Supplementary figures and images for: In vitro Synergy of Polyphenolic Extracts From Honey, Myrtle and Pomegranate Against Oral Pathogens, S. mutans and R. dentocariosa
Source: Front Microbiol. 2020 Jul 24;11:1465. doi: 10.3389/fmicb.2020.01465 (PMC7396681; doi:10.3389/fmicb.2020.01465)

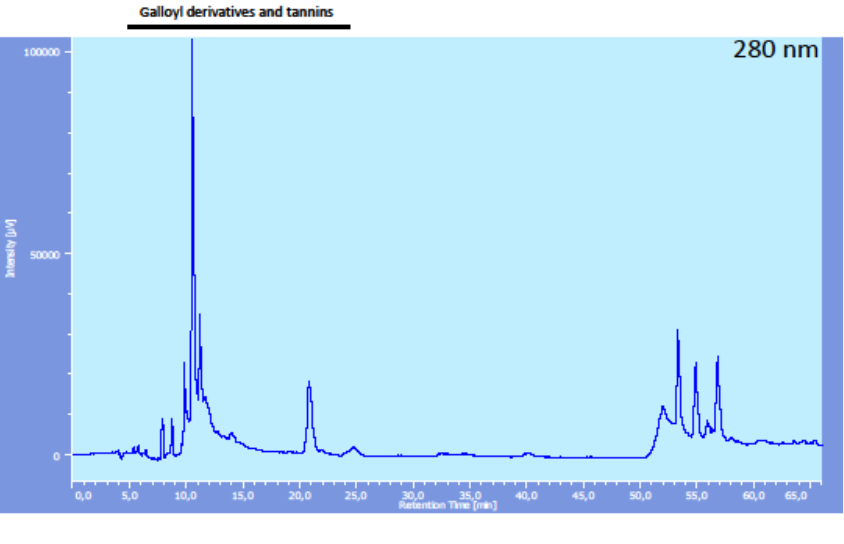

Supplement: FIGURE S1 — Chromatographic profile, acquired at 280 nm, of Myrtus communis L. leaves hydro-alcoholic extract. [file Image_1.TIF]
